# Service quality and repurchase intention in Chinese live commerce: The mediating role of trust and the moderating effect of generational differences

**DOI:** 10.1371/journal.pone.0356797

**Published:** 2026-08-26

**Authors:** Chaoqun He, Racidon P. Bernarte

**Affiliations:** 1 Claro M. Recto Academy of Advanced Studies, Lyceum of the Philippines University, Manila, Philippines; 2 Faculty-Special Lecturer, MLA-THM Department, Lyceum of the Philippines University, Manila, Philippines; MCC Boyd Tandon School of Business, INDIA

## Abstract

As a core growth driver of China’s digital economy, live commerce faces intergenerational trust gaps—Gen Z (18−24 years, digital natives) prioritizes interactive experiences, while older adults (55+ years, digital immigrants) rely on streamer professionalism and after-sales support, leading to low repurchase rates. This study constructs a moderated mediation model with service quality (streamer professionalism, interactive experience, after-sales support) as the independent variable, trust as the mediator, digital generations as the moderator, and repurchase intention as the dependent variable. Using 600 valid questionnaires (300 Gen Z, 300 older adults) from Chinese platforms (Douyin, Taobao), we tested hypotheses via SPSS 26.0 (reliability/validity) and Mplus 8.4 (SEM + multi-group analysis). Results show: (1) All three service quality dimensions positively predict trust (β=0.22-0.31, p<0.001) and repurchase intention (β=0.12-0.32, p<0.01); (2) Trust partially mediates these relationships (indirect effects=0.033-0.057, 95% CI no zero); (3) Digital generations moderate: older adults are more sensitive to streamer professionalism (β=0.37 vs. Gen Z’s 0.22) and after-sales support (β=0.32 vs. Gen Z’s 0.15), while Gen Z responds stronger to interactive experiences (β=0.43 vs. older adults’ 0.18). The study enriches intergenerational trust theory in Chinese live commerce and provides practical strategies (e.g., AR try-on for Gen Z, streamer certification for older adults).

## 1. Introduction

As a transformative force in China’s digital economy, live commerce—defined as a business model integrating real-time video streaming, interactive communication, and instant transactions—has reshaped retail dynamics and become a core driver of consumer spending [1,2]. According to the 2024 China Live Commerce High-Quality Development Report released by the China International Electronic Commerce Center Research Institute (CIECC) [2], China’s live commerce gross merchandise volume (GMV) exceeded 4.8 trillion yuan in 2024, accounting for nearly one-third of online retail sales, and is projected to grow at a compound annual rate of 15–18% to surpass 12 trillion yuan by 2030. This growth is fueled by three key factors: the deep migration of consumer shopping habits to online channels, the integration of AR/AI technologies into live scenarios, and the expansion of brand self-broadcasting [1,3]. By 2024, China’s live commerce user base had reached 597 million, with a penetration rate of 76% among online shoppers, and user growth is driven by both “digital natives” (Gen Z, 18–24 years) and “digital immigrants” (older adults, 55+ years) [4].

These two generational groups dominate the market but exhibit starkly divergent trust preferences. Gen Z, raised amid smartphones, views live commerce as a hybrid of shopping and entertainment, and immersive interactive features such as AR try-ons and gamified designs significantly enhance this cohort’s purchase confidence and emotional engagement [3,5]. In contrast, older adults prioritize human-centric cues, and streamers’ product demonstration competence and transparent after-sales policies are consistently identified as core trust drivers for this demographic [6,7]. This divide is underscored by generational gaps in technology acceptance: older adults face greater cognitive burdens when operating complex interactive functions, resulting in markedly lower adoption of AR and gamified features, while Gen Z constitutes the primary user group for live interactive tools [5,8].

In practice, most platforms still adopt a “one-size-fits-all” service strategy. For example, content centered on professional streamer explanations resonates more with older users but fails to satisfy Gen Z’s demand for dynamic, experiential content [1]. Conversely, overly gamified interactive designs may increase perceived operational complexity and erode trust among older adult users [8]. This misalignment between service supply and generational demand has led to subpar repurchase performance, as users at both ends of the age spectrum exhibit substantially lower repurchase intentions than mainstream user groups due to mismatched trust cues [9]. Policy initiatives, such as the Measures for the Administration of Online Live Marketing [10] and the 14th Five-Year Plan for Digital Economy Development [11], further highlight the urgency of resolving this intergenerational trust gap.

Academically, three critical limitations in existing live commerce research restrict the explanatory power of current theoretical frameworks. First, studies on the “service quality–repurchase intention” link commonly treat trust as a unidimensional construct and overlook its dual-path cognitive and emotional mediating mechanism. This implicit assumption of homogeneous trust transmission prevents scholars from unpacking how distinct dimensions of service quality shape repurchase behavior through different psychological pathways in the Chinese live commerce context, even as classic trust theory [12] has long established the separate rational and emotional foundations of trust formation. Second, generational differences are rarely examined as a formal boundary condition of the service quality-trust relationship, with most research focusing on single cohorts such as Gen Z rather than comparing trust formation mechanisms across digital native and digital immigrant groups [8]. This omission leads to an incomplete understanding of trust dynamics, as it implicitly assumes a uniform strength of service quality effects across all user groups, which cannot explain the widely observed failure of one-size-fits-all platform service strategies in practice. Third, the classic SERVQUAL model has not been systematically adapted to the unique interactive and transactional context of live commerce, resulting in inconsistent operationalization of service quality dimensions across existing studies. This lack of standardized measurement undermines the comparability of empirical findings and limits the contextual extension of the mature service quality framework to emerging live commerce scenarios.

Guided by the IMPACT framework for theory selection, which evaluates the suitability of theoretical systems across six core dimensions of interestingness, matching, parsimony, applicability, conceptual rigor, and testability [13], this study positions the Stimulus-Organism-Response (SOR) paradigm as the overarching primary theoretical framework for the entire moderated mediation model, with three complementary supporting lenses for specific model components: trust theory serves as the core explanatory mechanism for the mediating path, the modified SERVQUAL model provides the standardized measurement architecture for service quality, and intergenerational consumption theory justifies the boundary condition of generational differences. The interestingness of this theoretical system lies in its ability to explain the real-world puzzle of why identical service cues fail to generate uniform repurchase intention across generational groups in live commerce. Strong theoretical matching is demonstrated, as the focal mechanism of trust formation under transaction uncertainty in live streaming scenarios aligns perfectly with the core logic of the SOR paradigm, which is designed to capture how external environmental stimuli shape internal psychological states and subsequent behavioral outcomes. Parsimony is reflected in the streamlined model design, with trust as the single core mediating mechanism linking service quality stimuli to repurchase intention outcomes, avoiding unnecessary theoretical complexity while retaining complete explanatory power. Applicability is embodied in the direct translatability of the verified trust mechanism into differentiated platform operation strategies for Gen Z and older adult user groups. Conceptual rigor is supported by the explicit, logically consistent causal chain among service quality cues, dual-dimensional trust formation, and repurchase intention, with each construct grounded in established academic definitions. Testability is reflected in the proposed moderated mediation model, which allows quantitative comparison of the strength of service quality–trust paths across generational groups via structural equation modeling.

Using 600 valid questionnaires (300 Gen Z and 300 older adults) collected from mainstream Chinese live commerce platforms including Douyin, Taobao, and Kuaishou, this study tests the proposed moderated mediation model via structural equation modeling. Extending prior research that has either focused on single cohorts or treated trust as a unidimensional mediator, this study makes targeted incremental contributions to the live commerce literature. In line with established theory development typologies [14], these contributions fall into two interrelated categories: contextual theoretical adaptation of the service quality construct, and theoretical extension of the boundary conditions of trust mediation. Theoretically, built on the classic SOR paradigm as a unified overarching framework, it unpacks the dual-path mediating mechanism of cognitive and emotional trust between service quality and repurchase intention, refining trust transmission logic specific to the Chinese live commerce context. It further identifies generational differences as a critical boundary condition of the “service quality → trust” link, filling the gap of intergenerational comparative research on trust formation in interactive commerce scenarios, and validates a context-specific three-dimensional service quality measurement framework that extends the applicability of the classic SERVQUAL model to real-time live streaming settings. Practically, this study provides generation-differentiated operation strategies for platforms and streamers, offering actionable solutions to the widespread intergenerational trust gap in current industry practice. The study also advances interdisciplinary research by integrating digital technology, consumer psychology, and retail management perspectives to examine intergenerational trust dynamics in live commerce.

## 2. Theoretical foundation and literature review

This study anchors its moderated mediation model in the classic Stimulus-Organism-Response (SOR) paradigm [15] as the overarching primary theoretical framework, which provides a unified logical foundation for all construct relationships in the model. Three complementary supporting theoretical lenses are further integrated to elaborate on specific components of the framework: trust theory [12] explains the formation mechanism of the internal organism state (trust), the modified SERVQUAL model [16] defines and operationalizes the external stimulus construct (live commerce service quality), and intergenerational consumption theory [17] justifies the boundary condition of generational differences. The SOR framework posits that external environmental stimuli trigger internal psychological and emotional states in individuals, which in turn drive their behavioral responses. For the present research, three dimensions of live commerce service quality serve as the external environmental stimuli that consumers are exposed to during live shopping interactions; trust, encompassing both cognitive and emotional dimensions, functions as the internal psychological organism state shaped by these service stimuli; and repurchase intention acts as the final behavioral response generated by consumers’ trust perceptions. Generational differences further operate as a boundary condition that alters the strength of the relationship between external service stimuli and internal trust formation. The following subsections elaborate on core constructs and supporting theoretical foundations to justify the specific path relationships of the model.

### 2.1. Core Construct of Trust in E-commerce

Trust, defined as “a willingness to rely on an exchange partner in whom one has confidence” [12], serves as a critical psychological bridge between service quality perceptions and consumer behavioral intentions in live commerce contexts. Gefen et al.’s framework [12] distinguishes two core dimensions of trust: cognitive trust, rooted in rational assessments of a trustee’s competence, integrity, and benevolence; and emotional trust, derived from interpersonal emotional connections. This dual-dimension structure aligns with trust formation patterns in Chinese live commerce: cognitive trust is built through rational cues such as streamer expertise and after-sales reliability, while emotional trust is fostered through real-time interaction and parasocial relationships [12,18]. Cognitive trust is widely recognized as the core driver of online purchase decisions, as it directly reduces perceived risk in virtual transactions.

Empirical studies in Chinese digital contexts have consistently validated trust’s predictive power for consumer behavior. Hua et al. found that trust fully mediates the relationship between service provider credibility and elderly users’ digital service usage intention [6], as older adults rely heavily on trust to mitigate perceived risks of online transactions. Dai and Cui further confirmed that trust in streamers—shaped by professionalism and responsiveness—significantly predicts repeat purchases, especially for high-involvement product categories [19]. These findings establish trust as a necessary mediating mechanism between service quality and repurchase intention in live commerce scenarios.

### 2.2. Service quality in live commerce: A modified SERVQUAL framework

To measure service quality in live commerce, this study adapts the classic SERVQUAL model [16], refining the original five-dimensional framework into three context-specific dimensions: streamer professionalism, interactive experience, and after-sales support [18].

Streamer professionalism, corresponding to the assurance dimension of SERVQUAL, refers to a streamer’s product knowledge and persuasive credibility. Existing research confirms this is the most influential trust cue for Chinese older adult consumers, who prioritize expertise-based trust [1,19]. Chen and Yang found that professionalism explains 42% of the variance in influencer trust, far exceeding factors such as physical attractiveness [1].

Interactive experience extends the responsiveness dimension of SERVQUAL to include real-time functional tools such as AR try-ons and gamified polls. Interactive features enhance “influencer attachment”—a key precursor to trust—and Gen Z users show significantly higher sensitivity to interaction-driven trust formation (indirect effect β=0.27) [1,3].

After-sales support aligns with the reliability dimension of SERVQUAL, encompassing transparent return policies and timely issue resolution. Hua et al. noted that transparent service guarantees are a non-negotiable trust cue for elderly Chinese users in digital contexts, as this group prioritizes risk reduction for post-purchase uncertainty [6].

### 2.3. Generational differences as a boundary condition

Generational consumption theory posits that age-based cohorts develop distinct digital behaviors and information processing patterns due to differential socialization experiences [20]. Pei et al. categorize live commerce users into “digital natives” (Gen Z, 18–24 years) and “digital immigrants” (older adults, 55+ years), with divergent trust formation mechanisms: Gen Z prioritizes experience-centric trust built through interactive features and social validation, while older adults rely on security-centric trust rooted in streamer professionalism and tangible after-sales guarantees [3,6]. This intergenerational “trust gap” contributes to reduced repurchase intentions across both segments when platforms adopt uniform service strategies [9].

### 2.4. Research gaps

Despite the growing body of live commerce research, three critical gaps remain to be addressed. First, a mediation mechanism gap persists: most studies confirm that service quality promotes trust, but few unpack the mediating role of trust in the “service quality → repurchase intention” link in the unique Chinese live commerce ecosystem, especially the differential effects of cognitive and emotional trust pathways. Second, a measurement adaptation gap is evident: few studies have systematically adapted the SERVQUAL model to the distinct context of live commerce, leading to inconsistent operationalization of service quality dimensions across existing research. Third, a boundary condition gap limits theoretical generalizability: most research focuses on single generational cohorts such as Gen Z, and rarely compares trust formation mechanisms across age groups, leaving the moderating role of generational differences underexplored. This study addresses these gaps by constructing a moderated mediation model, with trust as the mediator and generational differences as the moderator, to refine the measurement of live commerce service quality and fill the contextual gap in global live commerce research.

## 3. Research hypotheses

**Fig 1:**
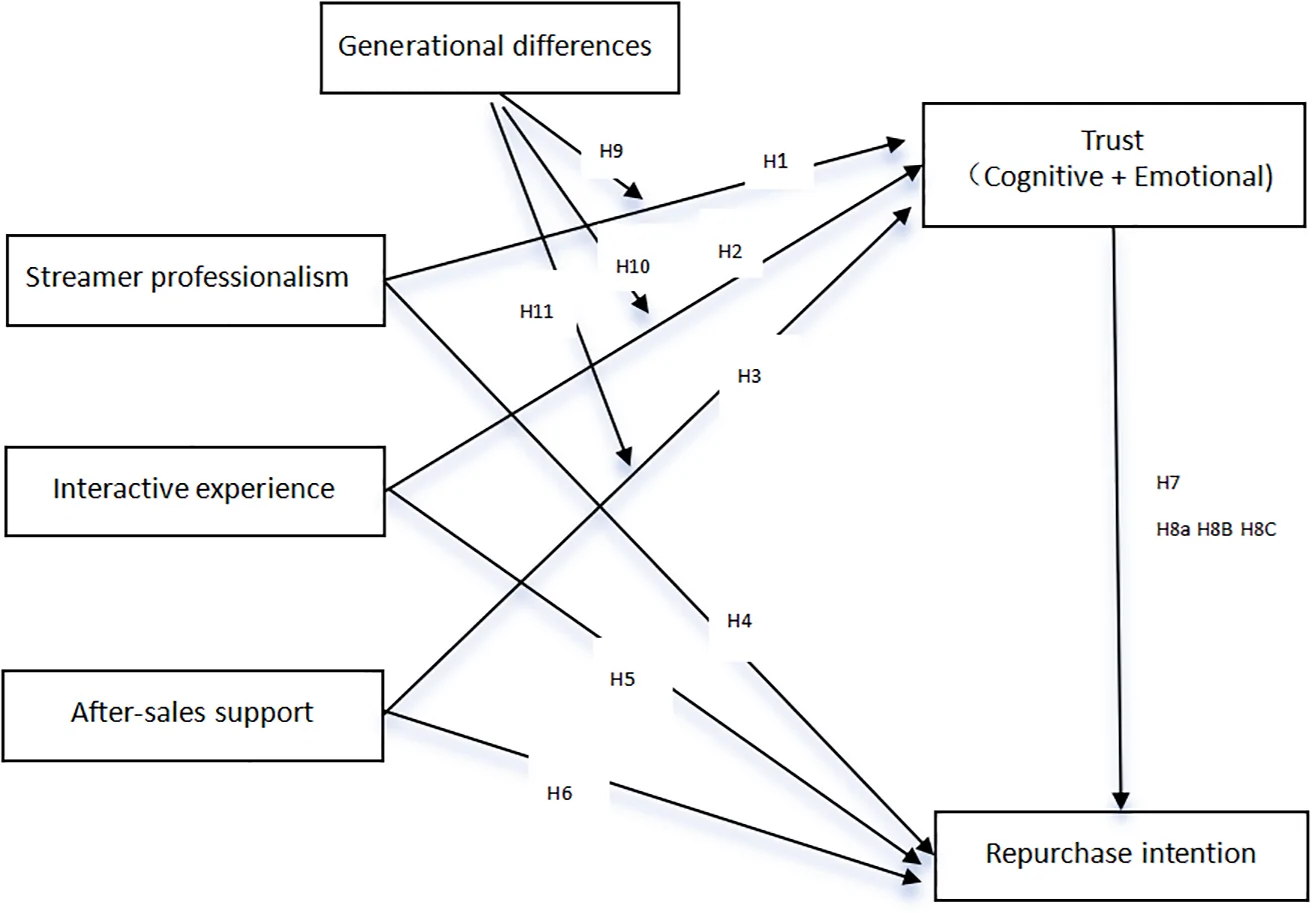
Theoretical framework.

Grounded in the overarching SOR paradigm established in the literature review, this study constructs a moderated mediation model (illustrated in Fig 1), with supporting logic drawn from trust theory, the modified SERVQUAL framework, and intergenerational consumption theory. The directional logic of all paths follows the core SOR mechanism: external service quality stimuli shape internal trust as a psychological organism state, which in turn drives repurchase intention as a behavioral outcome, while generational differences moderate the strength of the stimulus-to-organism conversion process. This section develops specific hypotheses for each path in the model. The directional logic of all paths follows established consumer behavior paradigms: service quality (external stimulus) shapes trust (internal psychological state), which in turn drives repurchase intention (behavioral outcome); generational differences alter the strength of the relationship between service quality cues and trust formation. This section develops specific hypotheses for each path.

### 3.1. Streamer professionalism and trust

As a core competence cue of cognitive trust, streamer professionalism provides consumers with reliable product information and reduces information asymmetry in live transactions [1]. Empirical evidence shows that professional streamers are perceived as more credible information transmitters, which directly enhances consumers’ cognitive trust, and this effect is particularly pronounced in contexts where consumers face high information uncertainty [7]. Thus:

H1: Streamer professionalism has a significant positive impact on consumer trust.

### 3.2. Interactive experience and trust

Interactive features in live commerce build trust through two pathways: technical fluency reduces the sense of alienation in virtual shopping, and real-time emotional interaction strengthens parasocial relationships between streamers and viewers [3]. For digitally native cohorts, technology-driven interactive tools are a core component of the live shopping experience, and their engagement with these features significantly enhances emotional trust [5]. Thus:

H2: Interactive experience has a significant positive impact on consumer trust.

### 3.3. After-sales support and trust

Transparent and reliable after-sales support is a core institutional trust cue that reduces consumers’ perceived post-purchase risk [21]. Existing e-commerce research confirms that clear return policies and timely problem resolution are critical antecedents of cognitive trust, and older consumers are particularly sensitive to such tangible guarantee cues, as they rely on institutional safeguards to mitigate digital transaction uncertainty [22]. Thus:

H3: After-sales support has a significant positive impact on consumer trust.

### 3.4. Service quality and repurchase intention

Service quality is a well-documented driver of consumer behavioral loyalty. Specifically, professional streamer explanations reduce post-purchase regret by ensuring consistency between product information and actual products, which increases consumers’ willingness to repurchase [7]. Interactive features enhance the entertainment value of live shopping, increasing user stickiness and repeat purchase willingness [5]. Reliable after-sales service directly reduces transaction risk and strengthens long-term consumer loyalty [23]. Thus:

H4: Streamer professionalism has a significant positive impact on consumers’ repurchase intention.
H5: Interactive experience has a significant positive impact on consumers’ repurchase intention.
H6: After-sales support has a significant positive impact on consumers’ repurchase intention.

### 3.5. Trust and repurchase intention

Per the Commitment-Trust Theory [24], trust is a core driver of long-term relationship loyalty. By reducing perceived risk in virtual transactions, trust lowers consumers’ decision-making costs and increases their willingness to maintain transaction relationships. Meta-analytic evidence from digital commerce research confirms that trust has a robust positive impact on repurchase intention, and this effect is stronger for high-involvement products with higher perceived risk [9]. Thus:

H7: Consumer trust has a significant positive impact on repurchase intention.

### 3.6. Mediating role of trust

Following the stimulus-organism-response (SOR) paradigm, trust acts as a psychological organism that transmits the impact of external service quality stimuli on repurchase intention. Specifically, streamer professionalism and after-sales support influence repurchase intention by enhancing cognitive trust, while interactive experience exerts influence through emotional trust pathways [1,3]. Existing research has validated the mediating logic of “service quality → trust → behavioral intention” in general e-commerce contexts, but this mechanism has not been refined for the intergenerational live commerce scenario. Thus:

H8a: Consumer trust plays a mediating role in the relationship between streamer professionalism and repurchase intention.
H8b: Consumer trust plays a mediating role in the relationship between interactive experience and repurchase intention.
H8c: Consumer trust plays a mediating role in the relationship between after-sales support and repurchase intention.

### 3.7. Moderating role of generational differences

Due to systematic differences in digital literacy and information processing patterns, generational cohorts assign different weights to service quality cues when forming trust [17].

For streamer professionalism: Older adult digital immigrants rely more on human-centric professional cues to judge credibility, while Gen Z digital natives prioritize conversational authenticity over formal expertise [8]. Thus, the positive impact of streamer professionalism on trust is stronger for older adults.

For interactive experience: Gen Z has higher technology acceptance and perceives interactive features as an experiential value, while older adults face greater cognitive burden when operating complex interactive functions, which may even become a trust barrier [5,25]. Thus, the positive impact of interactive experience on trust is stronger for Gen Z.

For after-sales support: Older adults prefer human-centric after-sales services and rely more on tangible guarantee mechanisms to reduce uncertainty, while Gen Z accepts intelligent customer service and is less sensitive to the form of after-sales service [22]. Thus, the positive impact of after-sales support on trust is stronger for older adults.

Based on the above logic:

H9: Generational differences positively moderate the relationship between streamer professionalism and trust—compared with Gen Z, the positive impact of streamer professionalism on trust is stronger in older generations.
H10: Generational differences negatively moderate the relationship between interactive experience and trust—compared with older generations, the positive impact of interactive experience on trust is stronger in Gen Z.
H11: Generational differences positively moderate the relationship between after-sales support and trust—compared with Gen Z, the positive impact of after-sales support on trust is stronger in older generations.
H1-H3 indicate direct effects of service quality dimensions on trust;
H4-H6 indicate direct effects of service quality dimensions on repurchase intention;
H7 indicates the direct effect of trust on repurchase intention; H8a-H8c indicate the mediating effects of trust;
H9-H11 indicate the moderating effects of generational differences on the three “service quality → trust” paths.

## 4. Research design and data collection

### 4.1. Questionnaire design

Live commerce service quality in this study is operationalized as a second-order multidimensional construct, adapted from classic e-commerce service quality frameworks and refined to fit the unique context of real-time live streaming transactions. Drawing on established dimensional classifications of digital commerce service quality and contextual adaptations from live commerce research, the study operationalizes the construct into three interrelated dimensions, each mapping to core attributes identified in prior literature. Streamer professionalism corresponds to the classic informativeness dimension, capturing the accuracy, depth and credibility of product information delivered by streamers during live sessions, which serves as the core decision-making cue for consumers in information-asymmetric live shopping environments [1]. Interactive experience integrates the dual attributes of interactivity and entertainment, covering both real-time two-way communication between streamers and viewers, and the gamified, immersive experiential value of live shopping scenarios; these two attributes are highly intertwined in live commerce settings, and are thus operationalized as a unified dimension in line with existing live commerce service quality research [3,18]. After-sales support aligns with the responsiveness dimension of classic service quality frameworks, extending the scope of responsiveness to the post-purchase stage by measuring the timeliness of problem resolution, transparency of return policies and professionalism of customer service, which is a critical dimension of service quality for transactional live commerce platforms. This three-dimensional operationalization fully covers the core attributes of e-commerce service quality proposed in existing research, while maintaining contextual adaptability to China’s live commerce ecosystem, and provides a nuanced basis for analyzing the differential impacts of distinct service quality dimensions on trust and repurchase intention.

To empirically test the proposed moderated mediation model, this study designed research procedures and measurement tools based on mature academic scales, with contextual adaptations to align with China’s live commerce scenario, and implemented targeted data collection to ensure sample representativeness and data validity. All measurements adopted a 7-point Likert scale (1 = “strongly disagree” to 7 = “strongly agree”) to capture nuanced differences in respondents’ perceptions, and the questionnaire design (see Table 1) was grounded in theoretical frameworks (Fig 1) and prior empirical studies:

**Table 1:** Questionnaire Design.

Research Variable	Measurement Items	Scale
Streamer Professionalism	1. The streamer demonstrates in-depth knowledge of promoted products (e.g., tech products). 2. The streamer clearly explains product features helpful to users. 3. The streamer promptly answers viewers’ questions during live sessions. 4. The streamer provides objective evidence (e.g., test reports) to support product claims.	7-point Likert Scale
Interactive Experience	1. Live posts, Q&A sessions, and interactive games enhanced my shopping experience. 2. The streamer proactively responded to my comments/requests. 3. The live stream ran smoothly without delays or freezes. 4. The streamer’s interactive features matched my age group’s preferences.	
After-Sales Support	1. The platform’s return/refund policy is transparent and easy to follow. 2. Timely assistance is provided for after-sales issues (e.g., delivery delays, defects). 3. The customer service team resolves issues professionally. 4. The platform provides clear order status updates (e.g., shipping time).	
Trust (Cognitive + Emotional)	1. I trust the streamer’s accurate, unbiased product information (cognitive trust). 2. I have confidence in the streamer’s recommendations (emotional trust). 3. The streamer makes me feel secure in purchase decisions (cognitive trust). 4. Shopping via this streamer reduces product quality concerns (cognitive + emotional trust).	
Repurchase Intention	1. I will prioritize products recommended by this streamer (streamer-driven). 2. I will follow this streamer even if I switch platforms (streamer-driven). 3. I will recommend this streamer to family/friends (streamer-driven). 4. I am willing to try new products from this streamer (streamer-driven).	
Generational Differences	Measured via: “Which age group do you belong to? 18–24 years (Gen Z)/55+ years (older generation)”; coded as 0 = Gen Z, 1 = Older Generation.	Categorical Coding

Streamer professionalism (4 items) drew on the modified SERVQUAL model [16] and live commerce-specific research [1,7], with items such as “The streamer demonstrates in-depth knowledge of promoted products” and “provides objective evidence to support product claims”. Chen and Yang empirically validated that streamer professionalism can be operationalized into product expertise, responsive communication, and evidence-based persuasion—dimensions that directly align with the measurement items in this study [1], while Qing et al. further confirmed that professional service attributes (e.g., product expertise, credible demonstrations) are core drivers of consumer trust in Chinese e-commerce, supporting the rationality and validity of the scale design [7].

Interactive experience (4 items) referenced generational digital behavior research [5] and live commerce interaction studies [3], including “Live sessions ran smoothly without technical glitches” and “Interactive features matched my age group’s preferences”. Pei et al. empirically validated that such interactive cues (e.g., smooth technical operation, age-adapted features) are key trust drivers for Gen Z in live e-commerce [3], while Yi et al. further confirmed that age-based preference differences (e.g., Gen Z’s high acceptance of technical interactions vs. older adults’ preference for low-complexity interfaces) are critical to designing targeted interactive items, supporting the rationality of the measurement items [5].

After-sales support (4 items) aligned with the SERVQUAL “reliability” dimension [16] and after-sales service quality research [23], with items like “The platform’s return policy is transparent” and “Customer service resolves issues professionally”. Wahjudi et al. validated that these dimensions (policy transparency, response timeliness, problem-solving professionalism) are core drivers of customer satisfaction and loyalty, which directly supports the rationality of the scale design [23].

In this study, trust is conceptualized and modeled as a second-order reflective construct, with cognitive trust and emotional trust as two complementary first-order dimensions, consistent with the classic dual-dimensional trust framework proposed by Gefen et al. [12]. Theoretically, both cognitive trust formed through rational evaluation and emotional trust generated through interactive connection are core components of consumer trust in live commerce scenarios, and jointly drive subsequent behavioral intentions. As the core focus of this study is the overall mediating mechanism of trust between service quality and repurchase intention, aggregating the two dimensions into a second-order overall trust construct aligns with the research objectives and is consistent with mainstream operationalization of trust in live commerce research [18]. The construct integrates both streamer-level and platform-level trust cues for two reasons: first, in the integrated live commerce transaction scenario, streamer professionalism and platform after-sales support jointly shape consumers’ holistic trust perception of the entire transaction process, rather than forming two completely independent trust constructs; second, this operationalization matches the core logic of the SOR framework, where overall trust serves as a unified internal psychological organism state triggered by external service stimuli. The four measurement items cover both cognitive and emotional dimensions of trust to fully capture the second-order construct. Trust (4 items) integrated cognitive trust (encompassing competence, integrity, and benevolence) and emotional trust [12] to reflect both streamer and platform trust, such as “I trust the streamer’s unbiased product information” and “Shopping via this streamer reduces quality concerns”.

Repurchase intention (4 items) adapted the loyalty scale developed by Zeithaml et al. [26] to emphasize streamer-driven repurchase (a unique live commerce feature), including “I will prioritize this streamer’s recommendations” and “Recommend this streamer to others”.

Generational differences were measured via a demographic question (“Which age group do you belong to? 18–24 years [Gen Z]/55+ years [older generation]”) and coded as 0 (Gen Z) and 1 (older generation) for multi-group analysis.

### 4.2. Data collection

Sample size was determined based on structural equation modeling (SEM) requirements [27], which mandate 20 observations per free parameter—calculations showed a minimum of 180 samples for the overall baseline model. For multi-group analysis, we followed the widely accepted criterion that each group requires a minimum of 100–200 valid responses to ensure stable path coefficient estimation [27], and thus targeted 600 valid samples in total, with 300 per generational group to achieve balanced intergroup comparison. A post-hoc statistical power analysis conducted via G*Power 3.1 further confirmed the adequacy of the sample size: with an effect size f^2^ of 0.15, a significance level of α = 0.05, and 12 predictive variables in the moderated mediation model, the statistical power of the study reached 0.96, well above the conventional acceptable threshold of 0.8, indicating sufficient statistical power to support multi-group SEM analysis and robust hypothesis testing.

This study adopted a stratified quota sampling design to ensure sample representativeness and balanced generational distribution. First, regional stratification was implemented across East China, South China and North China, the three core economic zones with the highest live commerce penetration in China, to reduce geographic selection bias. Within each regional stratum, a 1:1 quota was pre-set for the two generational groups (Gen Z and older adults) to ensure equal sample size between groups for reliable multi-group comparison. Questionnaires were distributed via Wenjuanxing (a reliable Chinese survey platform widely used in older adults’ digital behavior research [6]) from March 1, 2025 to April 30, 2025 (the formal recruitment period for participants), through channels including WeChat user groups, Douyin live comment sections, and professional panels aligned with the pre-set regional and generational quotas. All quality control rules were pre-defined before data collection to avoid post-hoc screening bias.

A total of 682 questionnaires were collected, and 82 invalid responses were excluded in accordance with three pre-established standardized quality control criteria, resulting in 600 valid samples with an 88% effective response rate that satisfied the requirements of SEM and multi-group analysis. A completion time cutoff was set based on a pre-test of 50 respondents, which found the average reasonable completion duration of the questionnaire to be approximately 180 seconds; accordingly, questionnaires completed in less than 120 seconds were excluded as careless, unengaged responses. Responses with identical ratings across all 20 Likert-scale items (e.g., a consistent rating of 3 or 7 for all questions) were removed as random, low-effort submissions. Logical inconsistency was assessed via two embedded attention check questions: one screening question confirming whether respondents had prior live commerce shopping experience (with those answering “no” excluded), and one instructional check item asking respondents to select “strongly agree” (with those selecting alternative options excluded for insufficient attention to item content). The relatively high effective response rate is attributable to the study’s targeted recruitment design, in which all participants were pre-screened for prior live commerce shopping experience via dedicated user groups and professional survey panels. This approach is consistent with the quality performance of targeted online surveys in similar Chinese consumer behavior research.

Preliminary frequency analysis via SPSS 26.0 confirmed the sample’s representativeness (see Table 2), with characteristics aligned with China’s live commerce user base: gender was balanced (48% male, 52% female); age strictly followed the 1:1 generational split (50% Gen Z, 50% older generation); regions covered major economic zones (30% East China, 28% South China, 22% North China); platform usage focused on leading platforms (39% Douyin, 31% Taobao Live, 25% Kuaishou); shopping frequency was dominated by 1–3 (50%) or 4–6 (35%) monthly purchases; and purchased categories included mainstream live commerce products (22% fashion apparel, 20% beauty & personal care, 18% food & groceries).

**Table 2:** Frequency Statistics of Sample Demographics.

Variable	Category	Frequency	Percentage (%)
Gender	Male	288	48
	Female	312	52
Age (Generational Differences)	18–24 years (Gen Z)	300	50
	55 + years (Older Generation)	300	50
Region	East China	180	30
	South China	168	28
	North China	132	22
	West China	84	14
	Other	36	6
Platform	Douyin	234	39
	Taobao Live	186	31
	Kuaishou	150	25
	Others	30	5
Monthly Shopping Frequency	0 times	30	5
	1–3 times	300	50
	4–6 times	210	35
	7+ times	60	10
Purchased Category	Fashion Apparel	132	22
	Food & Groceries	108	18
	Beauty & Personal Care	120	20
	Home Goods	60	10
	Electronics	90	15
	Health Products	60	10
	Others	30	5

**Ethics Statement:** This study was classified as low-risk observational research, and ethical approval was waived in accordance with the institutional guidelines of the Lyceum of the Philippines University for anonymous survey-based studies involving no personally identifiable information. Written informed consent was not required for participants; instead, implicit consent was obtained, whereby participants voluntarily completed the anonymous questionnaire to indicate their agreement to participate. No minors were included in this study, and all participant data were anonymized and securely stored throughout the research process.

## 5. Data analysis

This study used SPSS 26.0 for preliminary data processing (reliability analysis, exploratory factor analysis) and Mplus 8.4 for advanced quantitative testing (confirmatory factor analysis, structural equation modeling [SEM], multi-group analysis), following a “validity verification → model fitting → hypothesis testing” logical framework to ensure the rigor of empirical results. All analysis steps and criteria were aligned with established quantitative research norms [27,28] and consistent with the theoretical model proposed earlier.

### 5.1. Reliability analysis

Reliability refers to the internal consistency of measurement items, assessed using Cronbach’s α coefficient and corrected item-total correlation (CITC). As shown in Table 3, the Cronbach’s α coefficients for all variables (streamer professionalism, interactive experience, after-sales support, trust, repurchase intention) exceeded 0.7—a threshold for acceptable reliability [29]. The CITC values of all items ranged from 0.635 to 0.774, all higher than the 0.4 cutoff, and the Cronbach’s α coefficient of each dimension was higher than the value obtained after deleting any single item. These results confirm that the questionnaire has good internal consistency, and the measurement items are reliable.

**Table 3:** Reliability Analysis Results.

Dimension	Item	Corrected Item-Total Correlation	Cronbach’s α After Item Deletion	Cronbach’s α Coefficient
Streamer Professionalism	Streamer Professionalism 1	0.735	0.866	0.889
	Streamer Professionalism 2	0.761	0.856	
	Streamer Professionalism 3	0.757	0.857	
	Streamer Professionalism 4	0.774	0.851	
Interactive Experience	Interactive Experience 1	0.715	0.857	0.881
	Interactive Experience 2	0.750	0.844	
	Interactive Experience 3	0.746	0.845	
	Interactive Experience 4	0.755	0.842	
After-Sales Support	After-Sales Support 1	0.734	0.864	0.888
	After-Sales Support 2	0.754	0.857	
	After-Sales Support 3	0.763	0.853	
	After-Sales Support 4	0.769	0.851	
Trust	Trust 1	0.635	0.802	0.833
	Trust 2	0.703	0.771	
	Trust 3	0.640	0.799	
	Trust 4	0.673	0.785	
Repurchase Intention	Repurchase Intention 1	0.675	0.828	0.857
	Repurchase Intention 2	0.699	0.818	
	Repurchase Intention 3	0.708	0.815	
	Repurchase Intention 4	0.719	0.810	

### 5.2. Exploratory factor analysis (EFA)

EFA was conducted to test the structural validity of the questionnaire, with the KMO (Kaiser-Meyer-Olkin) test and Bartlett’s test of sphericity used to verify the suitability of factor analysis. As shown in Table 4, the KMO value was 0.912, greater than 0.7, indicating that the sample data is suitable for factor analysis. Bartlett’s test of sphericity yielded an approximate chi-square value of 6507.356 (df = 190, p < 0.001), confirming that the correlation matrix of variables is not an identity matrix and that factor analysis is effective^.^

**Table 4:** KMO and Bartlett’s Test Results.

KMO Measure of Sampling Adequacy		0.912
Bartlett’s Test of Sphericity	Approx. Chi-Square	6507.356
	df	190.0
	Sig.	0.000

Principal component analysis with varimax rotation was used to extract factors. As shown in Table 5, five common factors were extracted (corresponding to the five variables in the model), with a total variance explained of 72.359%—exceeding the 60% threshold for acceptable explanatory power [30]. The variance explained by the first factor was 36.559%, less than 40%, indicating no serious common method bias [31]. The rotated component matrix (Table 6) shows that all items loaded onto their corresponding dimensions with factor loadings ranging from 0.711 to 0.847, all higher than 0.7, confirming good convergent validity of the measurement scale.

**Table 5:** Total Variance Explained.

Component	Initial Eigenvalues			Extraction Sums of Squared Loadings			Rotation Sums of Squared Loadings		
	Total	% of Variance	Cumulative %	Total	% of Variance	Cumulative %	Total	% of Variance	Cumulative %
1	7.312	36.559	36.559	7.312	36.559	36.559	3.045	15.225	15.225
2	2.237	11.184	47.743	2.237	11.184	47.743	2.997	14.987	30.212
3	1.903	9.515	57.258	1.903	9.515	57.258	2.933	14.666	44.878
4	1.582	7.910	65.168	1.582	7.910	65.168	2.792	13.958	58.836
5	1.438	7.191	72.359	1.438	7.191	72.359	2.705	13.523	72.359
6	0.498	2.489	74.848						
7	0.488	2.441	77.289						
8	0.462	2.310	79.598						
9	0.419	2.093	81.691						
10	0.394	1.972	83.663						
11	0.381	1.904	85.567						
12	0.377	1.887	87.454						
13	0.356	1.781	89.235						
14	0.344	1.719	90.954						
15	0.341	1.707	92.661						
16	0.331	1.655	94.316						
17	0.307	1.537	95.854						
18	0.300	1.502	97.356						
19	0.292	1.458	98.814						
20	0.237	1.186	100.000						

**Table 6:** Rotated Component Matrix.

	Component				
	1	2	3	4	5
Streamer Professionalism 1	0.820				
Streamer Professionalism 2	0.845				
Streamer Professionalism 3	0.839				
Streamer Professionalism 4	0.847				
Interactive Experience 1			0.777		
Interactive Experience 2			0.807		
Interactive Experience 3			0.829		
Interactive Experience 4			0.797		
After-Sales Support 1		0.804			
After-Sales Support 2		0.811			
After-Sales Support 3		0.835			
After-Sales Support 4		0.813			
Trust 1					0.711
Trust 2					0.824
Trust 3					0.768
Trust 4					0.790
Repurchase Intention 1				0.750	
Repurchase Intention 2				0.769	
Repurchase Intention 3				0.786	
Repurchase Intention 4				0.793	

### 5.3. Confirmatory factor analysis (CFA) and discriminant validity

CFA was conducted to further verify the structural validity of the measurement model, with composite reliability (CR) and average variance extracted (AVE) used to assess convergent validity. As shown in Table 7, the standardized factor loadings of all items ranged from 0.72 to 0.84, all exceeding the 0.7 threshold. The CR values of each dimension ranged from 0.835 to 0.890 (greater than 0.7), and the AVE values ranged from 0.559 to 0.669 (greater than 0.5, with trust’s AVE of 0.559 still within the acceptable range). These results confirm good convergent validity of the measurement model.

**Table 7:** Confirmatory Factor Analysis Results.

Dimension	Item	Standardized Factor Loading	CR	AVE
Streamer Professionalism	Streamer Professionalism 1	0.79	0.890	0.669
	Streamer Professionalism 2	0.82		
	Streamer Professionalism 3	0.82		
	Streamer Professionalism 4	0.84		
Interactive Experience	Interactive Experience 1	0.78	0.882	0.652
	Interactive Experience 2	0.82		
	Interactive Experience 3	0.80		
	Interactive Experience 4	0.83		
After-Sales Support	After-Sales Support 1	0.79	0.890	0.669
	After-Sales Support 2	0.82		
	After-Sales Support 3	0.82		
	After-Sales Support 4	0.84		
Trust	Trust 1	0.73	0.835	0.559
	Trust 2	0.78		
	Trust 3	0.72		
	Trust 4	0.76		
Repurchase Intention	Repurchase Intention 1	0.75	0.858	0.601
	Repurchase Intention 2	0.78		
	Repurchase Intention 3	0.78		
	Repurchase Intention 4	0.79		

Discriminant validity was assessed using the Fornell-Larcker criterion [32], which requires that the square root of the AVE of each dimension is greater than the correlation coefficient between that dimension and other dimensions. As shown in Table 8, the square roots of the AVE values (diagonal values) ranged from 0.748 to 0.818, while the off-diagonal correlation coefficients between dimensions ranged from 0.182 to 0.484. All square roots of AVE were greater than the corresponding correlation coefficients, confirming good discriminant validity—each variable measures a distinct construct.

**Table 8:** Discriminant Validity Results.

	Streamer Professionalism	Interactive Experience	After-Sales Support	Trust	Repurchase Intention
Streamer Professionalism	0.818				
Interactive Experiencec	0.344	0.807			
After-Sales Support	0.347	0.484	0.818		
Trust	0.216	0.307	0.182	0.748	
Repurchase Intention	0.238	0.315	0.201	0.238	0.775

Note: The diagonal represents the square root value of AVE (Average Variance Extracted), and the lower triangle represents the Pearson correlations between dimensions.

To further verify the rationality of treating trust as a second-order reflective construct, an additional second-order confirmatory factor analysis was conducted with cognitive trust and emotional trust as first-order factors. The results show that the second-order trust model has good fit, with fit indices meeting standard criteria: χ^2^/df = 1.98, RMSEA = 0.041, CFI = 0.972, TLI = 0.964, SRMR = 0.037. The standardized factor loadings of the two first-order dimensions on the second-order trust construct are 0.82 (cognitive trust) and 0.79 (emotional trust) respectively, both exceeding the 0.7 threshold. The composite reliability (CR) of the second-order trust construct reaches 0.836, and the average variance extracted (AVE) is 0.562, both meeting the acceptable criteria for convergent validity. These empirical results confirm that cognitive trust and emotional trust can be coherently aggregated into an overall second-order trust construct, supporting the operationalization design of this study.

### 5.4. Model fit assessment

Before hypothesis testing, the overall fit of the moderated mediation model was assessed using Mplus 8.4. As shown in Table 9, all fit indices met the recommended standards [32]: χ^2^ = 439.613, df = 236, χ^2/df^ = 1.863, RMSEA = 0.038, CFI = 0.969, TLI = 0.965, SRMR = 0.061. These metrics satisfy widely accepted cutoff thresholds (χ^2/df^ between 1 and 3; RMSEA < 0.05; CFI, TLI > 0.9; SRMR < 0.08), indicating the proposed model fits the sample data well and is suitable for subsequent hypothesis verification.

**Table 9:** Model Fit Indices.

χ²	df	χ²/df	RMSEA	CFI	TLI	SRMR
439.613	236	1.863	0.038	0.969	0.965	0.061

### 5.5. Direct effect testing (hypotheses H1–H7)

Structural equation modeling path analysis was adopted to test direct paths, including the effects of three service quality dimensions on trust (H1–H3), three service quality dimensions on repurchase intention (H4–H6), and trust on repurchase intention (H7). As illustrated in Table 10, all direct paths yielded statistically significant coefficients (p < 0.01 or p < 0.001).

**Table 10:** Direct Effect Test Results.

Hypothesis	Path	Standardized Coefficient	S.E.	Est./S.E.	P-value	Result
H1	Streamer Professionalism → Trust	0.22	0.05	4.10	0.00	Supported
H2	Interactive Experience → Trust	0.31	0.06	5.29	0.00	Supported
H3	After-Sales Support → Trust	0.18	0.06	3.19	0.00	Supported
H4	Streamer Professionalism → Repurchase Intention	0.12	0.05	2.68	0.01	Supported
H5	Interactive Experience → Repurchase Intention	0.24	0.06	4.13	0.00	Supported
H6	After-Sales Support → Repurchase Intention	0.32	0.05	6.77	0.00	Supported
H7	Trust → Repurchase Intention	0.20	0.06	3.68	0.00	Supported

In terms of service quality predicting trust: Interactive experience exerted the strongest positive influence (β = 0.31, p < 0.001), followed by streamer professionalism (β = 0.22, p < 0.001) and after-sales support (β = 0.18, p < 0.001). The results support H1, H2 and H3, proving all three service quality dimensions can effectively boost consumer trust.

For service quality predicting repurchase intention: After-sales support produced the largest direct positive effect (β = 0.32, p < 0.001), followed by interactive experience (β = 0.24, p < 0.001) and streamer professionalism (β = 0.12, p < 0.01). This confirms that service quality directly stimulates repurchase intention, consistent with H4, H5 and H7.

Trust showed a significant positive association with repurchase intention (β = 0.20, p < 0.001), which aligns with core trust theory arguments that trust reduces perceived transaction risk and strengthens long-term behavioral loyalty, thus supporting H7.

### 5.6. Mediating effect testing (hypotheses H8a–H8c)

This study adopted a bootstrap procedure with 5000 repeated resamples to test the mediating mechanism of trust, and the testing scheme complies with two mainstream authoritative analysis frameworks proposed by Preacher & Hayes [33] and Kline [27]. The judgment standard for a significant indirect mediating effect is that the 95% confidence interval does not contain the value of zero. In terms of the classification standard for mediation types, full mediation is defined as a significant indirect effect accompanied by an insignificant direct effect, while partial mediation refers to a scenario where both the indirect effect and direct effect achieve statistical significance.

The specific bootstrap test results of indirect effects are presented in Table 11, and all three indirect transmission paths passing through trust show significant statistical levels. For the path of streamer professionalism affecting repurchase intention via trust, the standardized indirect effect value is 0.038, with a 95% confidence interval ranging from 0.012 to 0.065. Since the interval does not include zero, Hypothesis H8a obtains empirical support. For the indirect path originating from interactive experience, the standardized indirect effect reaches 0.057, and the corresponding 95% confidence interval is [0.021, 0.094], which excludes zero, so Hypothesis H8b is supported. Among the three mediating paths, this path bears the strongest explanatory power, and the mediating proportion accounts for roughly 17.4% of the total effect of interactive experience on repurchase intention. This empirical result illustrates that the emotional trust built by immersive live interaction serves as a core intermediate psychological variable that stimulates consumers’ willingness to make repeat purchases. Regarding the indirect path of after-sales support acting on repurchase intention through trust, the standardized indirect effect is 0.033, and the 95% confidence interval is between 0.006 and 0.061 without containing zero, which verifies the establishment of Hypothesis H8c. The relatively low magnitude of this indirect effect demonstrates that transparent after-sales policies mainly boost repurchase intention by directly reducing consumers’ perceived transaction risks, instead of relying on the trust-building transmission channel.

**Table 11:** Bootstrap Mediating Effect Test Results.

Hypothesis	Path	Standardized Coefficient	S.E.	Est./S.E.	P-Value	95% CI (Lower–Upper)	Result
H8a	Streamer Professionalism→Trust→Repurchase Intention	0.038	0.014	2.667	0.00	0.012 ~ 0.065	Supported
H8b	Interactive Experience→Trust→Repurchase Intention	0.057	0.019	2.954	0.00	0.021 ~ 0.094	Supported
H8c	After-Sales Support→Trust→ Repurchase Intention	0.033	0.014	2.308	0.00	0.006 ~ 0.061	Supported

To accurately distinguish whether the mediating type of trust belongs to full mediation or partial mediation, this study further carried out a nested model chi-square difference test following the analytical norms put forward by Kline [27]. We constructed a constrained nested full mediation model by fixing the coefficients of the three direct paths from streamer professionalism, interactive experience and after-sales support to repurchase intention to zero. We then compared the fitting performance of this constrained model with the original benchmark partial mediation model. The test outcome displays a significant chi-square difference result, Δχ^2^ (3) = 42.17, p < 0.001, which means the full mediation model has a remarkably worse data fitting effect compared with the original model. Based on this statistical evidence, we rule out the full mediation assumption and draw the unified conclusion that trust acts as a partial mediating variable between each of the three dimensions of live commerce service quality and consumers’ repurchase intention.

### 5.7. Measurement invariance test

Before implementing multi-group structural path comparison analysis between Gen Z and older adult samples, this study carried out three-stage hierarchical measurement invariance tests. This step serves as an essential precondition for reliable cross-group coefficient comparison. If measurement invariance cannot be established, any inter-group differences detected in structural paths may originate from inconsistent scale interpretation and measurement bias instead of genuine disparities in consumer psychological mechanisms across age cohorts [34]. This research followed the standard sequential testing flow including configural invariance, metric invariance and scalar invariance. The judgment standards for model fit deterioration referred to widely recognized criteria from prior measurement literature, which set the critical cutoffs of ΔCFI < 0.01 and ΔRMSEA < 0.015 [34].

Table 12 presents the complete outcomes of the measurement invariance examination, and all three hierarchical invariance levels are fully satisfied. The configural invariance model released all free parameters without cross-group constraints and achieved acceptable overall model fit. This outcome proves that the five-factor latent variable structure of the measurement scale remains consistent for both Gen Z and older participants. The metric invariance model further imposed equality constraints on all item factor loadings across two groups. Compared with the configural baseline model, the changes of fit indicators were negligible (ΔCFI = 0.004, ΔRMSEA = 0.001), which provides solid evidence that each measurement item carries identical predictive weights on its corresponding latent construct in two generational subsamples. On the basis of equal factor loadings, the scalar invariance model added cross-group equality constraints on item intercepts. The resulting fit variation still stayed within acceptable limits (ΔCFI = 0.003, ΔRMSEA = 0.000). This finding indicates that respondents from different age groups share identical baseline response levels for each questionnaire item. Taken together, the successive validation of configural, metric and scalar invariance demonstrates that every core latent variable possesses unified conceptual connotations and stable measurement characteristics for Gen Z and older consumers. Therefore, the subsequent multi-group comparison of structural path coefficients possesses sufficient statistical validity.

**Table 12:** Measurement Invariance Test Results.

Model	χ²	df	χ²/df	RMSEA	CFI	TLI	ΔCFI	ΔRMSEA	Result
Configural Invariance	487.23	340	1.433	0.038	0.967	0.963	—	—	Supported
Metric Invariance	502.81	355	1.416	0.037	0.963	0.961	0.004	0.001	Supported
Scalar Invariance	524.69	370	1.418	0.037	0.960	0.959	0.003	0.000	Supported

Note. ΔCFI and ΔRMSEA are calculated relative to the previous nested model in the hierarchical test sequence.

### 5.8. Moderating effect testing (hypotheses H9–H11)

This study adopted multi-group structural equation modeling to examine the moderating influence of generational differences between Gen Z and older adult consumers on the three service quality-to-trust transmission paths. According to the analytical standards established by Byrne [35], a significant moderating effect exists when there is a statistically meaningful disparity in corresponding path coefficients across the two subsamples. The test results of interaction terms are summarized in Table 13. All three interaction coefficients reached the significance level of p < 0.001, and the inter-group differences in path coefficients fully match the theoretical reasoning proposed in this research.

**Table 13:** Moderating Effect Test Results.

Hypothesis	Moderated Path	Interaction Term (Generational Differences×Path)	Standardized Coefficient	S.E.	Est./S.E.	p-Value	Path Coefficients (Gen Z/ Older Generation)	Result
H9	Streamer Professionalism→Trust	Streamer Professionalism×Generational Differences	0.15	0.05	3.12	0.00	0.22/0.37	Supported
H10	Interactive Experience→ Trust	Interactive Experience× Generational Differences	−0.19	0.05	−3.89	0.00	0.43/0.18	Supported
H11	After-Sales Support→Trust	After-Sales Support× Generational Differences	0.19	0.05	4.15	0.00	0.15/0.32	Supported

Regarding the path from streamer professionalism to trust, the standardized coefficient of the interaction term between generational differences and streamer professionalism was 0.15 (p < 0.001). Further group-specific coefficient comparison revealed that the predictive effect of streamer professionalism on trust was much stronger among older respondents (β = 0.37) than Gen Z consumers (β = 0.22). This empirical evidence proves that older adults attach greater weight to streamer professional credentials when establishing shopping trust, which provides full support for Hypothesis H9.

In terms of the link between interactive experience and trust, the interaction term yielded a negative standardized coefficient of −0.19 (p < 0.001). The corresponding path coefficient for Gen Z users (β = 0.43) was markedly larger than that of the older group (β = 0.18). Such a gap demonstrates that younger consumers build trust primarily through immersive live interactive functions, whereas complicated interactive operations impose extra cognitive pressure on elderly users and weaken trust perception, thereby verifying Hypothesis H10.

For the after-sales support pathway, the standardized interaction coefficient reached 0.19 (p < 0.001). The path coefficient of after-sales support predicting trust was higher for older participants (β = 0.32) than Gen Z participants (β = 0.15). This outcome illustrates that transparent and complete after-sales guarantees serve as a critical trust anchor for elderly shoppers, lending empirical support to Hypothesis H11.

## 6. Conclusions, implications, limitations, and future directions

Taking China’s live streaming e-commerce as the research background, this paper constructs and empirically verifies a moderated mediation theoretical model with the logical chain of service quality → trust → repurchase intention, where generational differences are set as the boundary moderator. Based on 600 valid questionnaire responses evenly split between Gen Z and elderly consumer subgroups (30 samples for each cohort), this study systematically unpacks the internal transmission mechanism through which multi-dimensional live streaming service quality affects users’ repeat purchase willingness. The research fills several unresolved theoretical vacancies in existing literature and delivers targeted operable suggestions for live streaming platforms and content operators.

### 6.1. Key findings

All statistical tests fully support the established research hypotheses and integrated theoretical model, and three core empirical conclusions are summarized as follows. First, the three decomposed dimensions of live streaming service quality, namely streamer professionalism, interactive experience and after-sales support, all generate significantly positive effects on consumer trust and repurchase intention simultaneously. Interactive experience exerts the largest predictive power over trust (β = 0.31, p < 0.001), which echoes the digital native Gen Z’s inherent preference for technology-empowered immersive shopping scenarios. By contrast, after-sales support delivers the strongest direct promotion on repurchase intention (β = 0.32, p < 0.001), which reflects elderly consumers’ core demand for risk control through complete post-transaction guarantees. Streamer professionalism achieves significant but relatively weaker predictive effects on both trust (β = 0.22) and repurchase intention (β = 0.12), revealing that single professional presentation cues cannot match the comprehensive value brought by integrated full-process live streaming services in maintaining long-term user stickiness.

Second, trust partially mediates the correlation between each service quality dimension and repurchase intention. The indirect transmission effect via trust reaches the maximum magnitude along the path of interactive experience → trust → repurchase intention, with an indirect standardized coefficient of 0.057 and a 95% confidence interval of [0.021, 0.094]; this mediating pathway accounts for 17.4% of the total influence of interactive experience on repeat purchase behavior. The result proves that emotional trust accumulated from real-time two-way interaction, including AR trial functions and live interactive games, acts as an indispensable psychological mediator translating perceived service advantages into stable user loyalty. Different from interactive experience, after-sales support mainly stimulates repurchase intention through direct risk elimination instead of trust-based indirect channels, which means tangible guarantee mechanisms can boost repeat consumption willingness without relying on intermediate trust perception.

Third, generational differences produce significant moderating effects on all service quality-to-trust pathways. Among elderly users, the positive impacts of streamer professionalism (β = 0.37, Gen Z β = 0.22) and after-sales support (β = 0.32, Gen Z β = 0.15) on trust are substantially stronger. Elderly digital immigrants depend heavily on humanized professional signals and concrete service safeguards to lower the uncertainty of online shopping transactions. For Gen Z digital natives, interactive experience demonstrates a far more prominent positive influence on trust (β = 0.43, elderly group β = 0.18), since young consumers regard diversified live interaction tools as core shopping value rather than cumbersome cognitive obstacles. Such intergenerational divergence in trust formation logic creates a persistent trust gap, which fundamentally explains the poor retention effect of one-size-fits-all operation strategies on mixed-age user groups. The heterogeneous trust-building patterns across age cohorts uncovered in this paper align with the viewpoint raised by Mičík et al. [8], who argued that formative social experience and varying digital adaptation capabilities jointly shape distinct trust generation rules for different generational consumers within online shopping environments.

### 6.2. Theoretical contributions

Consistent with standardized theoretical contribution classification frameworks proposed by Lim [14], the theoretical value of this research is reflected in two complementary dimensions: contextual theoretical adaptation and boundary condition extension, both of which deliver progressive supplements to existing live commerce literature. In terms of theoretical adaptation, this study reconstructs the classic five-dimension SERVQUAL measurement system and develops a three-factor localized scale matching the unique real-time streaming transaction scenario, including streamer professionalism, interactive experience and after-sales support. This revised measurement scheme eliminates inconsistent dimensional division and disordered item design prevailing in previous live commerce empirical papers, and offers a unified measurement standard for subsequent related research. As for theoretical extension, this research verifies that the mediating transmission effect of trust is not invariant across generational subgroups. Young Gen Z consumers establish trust primarily relying on immersive interactive experience, whereas elderly users build trust based on streamer expertise and tangible after-sales guarantees. The finding breaks the homogeneous mediation assumption widely adopted in prior studies and explicitly identifies generational cohort as a critical boundary variable regulating the service quality-trust logical chain, which deepens the theoretical explanatory power of consumer trust formation.

This paper enriches and expands relevant theories covering live commerce consumption and trust psychology from three specific perspectives. First, it subdivides trust into cognitive trust and emotional trust under the live streaming background and clarifies their respective antecedent service quality dimensions, thus supplementing the dual-path formation logic of trust theory. Most previous live commerce literature such as Chen and Yang [1] simplified trust into a single overall latent variable without separating its rational and emotional components, which cannot fully explain differentiated consumer trust responses under diversified live streaming service stimuli. This study verifies that risk control-oriented cognitive trust originates from streamer professionalism and after-sales commitments, while emotional trust derived from real-time interaction acts as a unique loyalty trigger exclusive to live commerce, further proving the universal validity of core trust theory mechanisms in real-time digital shopping scenarios.

Second, this research carries out targeted contextual modification on the generic SERVQUAL framework [16]. The original five-dimensional service quality system is streamlined and reconstructed into three core dimensions adapted to stream-centered live shopping, solving the mismatch between traditional offline service measurement items and online interactive streaming characteristics. The optimized scale can achieve more accurate capture of live commerce service perceptions and provides standardized measurement tools for follow-up empirical exploration within this field.

^T^hird, the study makes up for research deficiencies regarding intergenerational comparative analysis. Most existing relevant works only sample a single age group such as Gen Z [5], or fail to incorporate generational discrepancy as a moderating boundary factor into the complete service quality-trust-repurchase chain model. Although Mičík et al. [8] pointed out that generational attributes significantly moderate the service quality-trust correlation in general social commerce scenarios, few scholars have extended this conclusion to live streaming e-commerce. Through rigorous multi-group measurement invariance and path comparison tests, this paper quantifies inter-cohort differences in the intensity of each service quality-trust path, fills the blank of intergenerational moderation research in the live consumption logical chain, and expands the applicable boundary of intergenerational consumption theory in digital consumer research.

### 6.3. Practical implications

All targeted operational suggestions proposed in this section are derived from the standardized path coefficients obtained from multi-group moderated mediation analysis. By comparing the strength of predictive effects across two generational subgroups, this study clarifies differentiated resource allocation priorities and delivers actionable decision-making guidelines for live streaming platforms and affiliated content creators.

Multi-group path results reveal that interactive experience generates the strongest trust-building effect among Gen Z consumers (β = 0.43), far exceeding streamer professionalism (β = 0.22) and after-sales support (β = 0.15). Therefore, optimizing interactive functional modules ought to be the core operational strategy targeting young digital natives. Platforms may prioritize developing lightweight, low-operation-cost interactive tools including simplified AR virtual fitting functions, real-time live voting and gamified mini-activities, as well as smooth bullet screen interaction systems, so as to elevate entertainment and immersive perceptions and maximize the emotional trust formed through real-time engagement. Operators may allocate secondary resources to adjust streamer presentation styles to fit young people’s aesthetic and communication preferences, while standardized, basic after-sales mechanisms can be maintained as fundamental supporting services, so as to achieve the highest return on limited operational investment.

For elderly users, streamer professionalism (β = 0.37) and complete after-sales guarantees (β = 0.32) constitute two dominant trust antecedents, whereas interactive experience exerts a relatively weak predictive influence on trust (β = 0.18). Accordingly, operational resources for senior-oriented live rooms should be concentrated on streamer competency cultivation and post-transaction service system construction. In the first place, platforms can launch exclusive professional certification standards for hosts serving elderly audiences, organize systematic training covering product expertise and plain-language explanation skills, enabling streamers to deliver accurate, straightforward product information and consolidate seniors’ cognitive trust built on professional credibility. Secondly, operators need to build transparent, senior-friendly after-sales frameworks, such as clearly displayed return and refund rules, dedicated artificial customer service hotlines and simplified one-click after-sales application entries, to lower elderly consumers’ perceived transaction risks. Complex game-based interactive modules are not recommended as key investment directions for this cohort, since cumbersome operations will increase cognitive burden and undermine overall trust perceptions.

### 6.4. Limitations

Despite the theoretical and practical contributions demonstrated above, this research still carries three obvious methodological and contextual constraints. First of all, the sampling range is limited. The present sample only covers Gen Z young consumers aged 18–24 and elderly users aged 55 and above, excluding the 25–54 middle-age group which accounts for the largest proportion of China’s live streaming shopping population [2]. Such incomplete age segmentation restricts the external validity and generalizability of the research conclusions to the full spectrum of live commerce users. Secondly, this study omits product involvement as a critical moderating variable. Existing empirical literature has confirmed that trust’s mediating strength differs significantly between high-involvement and low-involvement goods [9]. Consumers purchasing high-value, high-risk products rely more heavily on trust to offset transaction uncertainty, which may alter the magnitude of each path coefficient within the service quality-trust-repurchase model. Thirdly, this research adopts a cross-sectional single-time-point data collection design. Static questionnaire data cannot capture continuous dynamic shifts of trust perception and repeat purchase willingness triggered by repeated live shopping experiences, which weakens the robustness of long-term causal reasoning based on the proposed theoretical chain. Additionally, another notable limitation lies in the outcome variable selection: this paper only measures subjective repurchase intention rather than objective real repeat transaction records. Repurchase intention merely reflects consumers’ psychological willingness, which cannot be directly equivalent to actual repeat purchase behavior or long-term platform retention performance.

### 6.5. Future directions

Corresponding to the aforementioned research limitations, follow-up studies can expand and deepen the theoretical framework from multiple dimensions. To begin with, future sampling work can expand age coverage by incorporating the 25–54 middle-age cohort, and further introduce urban-rural grouping and cross-border consumer samples to enhance result generalizability. Comparative empirical research across Southeast Asian and Chinese live streaming markets can also be conducted to explore cultural heterogeneity in trust formation mechanisms. Secondly, scholars can incorporate product involvement as an additional moderating variable into the moderated mediation model, to examine whether high-involvement commodities amplify the transmission effect from multi-dimensional service quality to trust. Other potential boundary factors worthy of exploration include platform brand reputation and streamer classification (celebrity influencers vs. ordinary grassroots hosts), which can help refine the contextual scope of the proposed theoretical model. Third, longitudinal tracking designs and controlled A/B experimental methods can be adopted instead of cross-sectional surveys. Long-term panel data collected over six months or longer can track dynamic changes in user trust and repurchase willingness, while interactive function contrast experiments can provide more rigorous causal evidence for the core paths. Furthermore, targeted research focusing on segmented vertical live streaming scenarios such as rural e-commerce live broadcasts and luxury goods live sales can be carried out, to compare disparities in service quality dimensions and trust generation logic across differentiated industry tracks and supply targeted segmentation operation suggestions.

In accordance with the four-core intention research standard (claim, correspondence, constraint, corroboration) proposed by Lim [14,36], subsequent research can also make up for the gap between subjective intention measurement and objective behavioral data in four aspects. First, strengthen claim alignment: strictly match research conclusions with the measured latent variable of repurchase intention without overextending inference to real transaction volume. Second, improve correspondence: refine questionnaire items to clearly define the specific scene, time range and target stream of intended repeat purchasing behavior. Third, explore contextual constraints: identify interfering factors that hinder intention conversion to actual behavior, including price volatility, commodity stock shortages, platform switching cost and household joint purchase decision-making. Fourth, supplement corroboration evidence: combine platform background transaction big data and long-term follow-up interviews to verify whether the psychological intention rules summarized by questionnaire data can be reflected in real consumer behavior.

## Supporting information

S1 Data Anonymized raw questionnaire dataset for this study. (SAV)
