# Supplementary material for: Service quality and repurchase intention in Chinese live commerce: The mediating role of trust and the moderating effect of generational differences
Source: PLoS One. 2026 Aug 26;21(8):e0356797. doi: 10.1371/journal.pone.0356797 (PMC13514050; doi:10.1371/journal.pone.0356797)
Supplement: Supplementary file 1 [file pone.0356797.s003.docx]

**Response to Reviewers: Service Quality and Repurchase Intention in Chinese Live Commerce**

**Response to Reviewers**

Manuscript ID: PONE-D-26-06405
Title: Service Quality and Repurchase Intention in Chinese Live Commerce: The Mediating Role of Trust and the Moderating Effect of Generational Differences
Corresponding Author: Chaoqun He
Submission Date: June 12, 2026

**Preface**

We sincerely appreciate the Academic Editor and all reviewers for their detailed, constructive feedback, which significantly improves the theoretical rigor, methodological transparency, writing standard and practical value of this manuscript. We have fully addressed every comment point-by-point in the following text. All revisions are marked with track changes in the uploaded revised manuscript file. All formatting adjustments strictly comply with official PLOS ONE submission standards, including reference normalization, table/figure specifications, data availability and ethics statement requirements.

**Part 1 Responses to Academic Editor’s Mandatory Comments**

**Editor Comment 1: Non-academic sources (Renrendoc, Originality Documents, Docin) cannot be cited; replace with credible peer-reviewed or official statistical sources**

**Response**

We fully agree and have completed comprehensive revisions.

1. All citations to commercial document-sharing platforms (Renrendoc, Originality Documents, Docin) are completely deleted throughout the full text.
2. All market scale, user demographic and live commerce behavioral statistics are replaced with authoritative, traceable primary sources:

- Official national reports: *The 55th Statistical Report on China’s Internet Development* (CNNIC, 2025), *2024 China Live Commerce High-Quality Development Report* (China International Electronic Commerce Center, 2025);
- Peer-reviewed empirical papers published 2023–2025 focusing on Chinese live commerce;
- Authoritative third-party industry white papers with open academic archives.

3. Every statistical claim in the Introduction and Literature Review is attached a formal citation with complete DOI. All data can be traced to original official documents, eliminating reliance on uncertified grey commercial literature.

**Editor Comment 2: Line 70 contains an impossible figure “China’s live commerce user base exceeded 8.2 billion by 24”; revise the numerical error**

**Response**

This mistake arises from a unit translation typo, and we have fully corrected and cross-verified the data with official CNNIC 2025 reports.

- Original erroneous statement: 8.2 billion live commerce users
- Revised formal statement: By 2024, China’s live commerce active users reached 597 million, with a penetration rate of 76% among all Chinese online shoppers (China Internet Network Information Center, 2025).
  We added a brief explanatory note in the Introduction to clarify the previous unit error, and updated all related user proportion statistics consistently across the whole manuscript.

**Editor Comment 3: The authors claim trust acts as a partial mediator without formal statistical comparison between full and partial mediation models**

**Response**

We supplemented two authoritative statistical tests to formally verify mediation type and added relevant results to Section 5.6 Mediating Effect Testing and Table 11:

1. Bootstrap joint significance test (Preacher & Hayes, 2008): All three indirect paths (service quality → trust → repurchase intention) and all three direct service quality → repurchase paths show significant 95% CIs excluding zero, satisfying the statistical standard for partial mediation.
2. Nested model chi-square difference test (Kline, 2023): We constructed a constrained full mediation model (all direct service quality-repurchase paths fixed at 0) and compared it with the baseline partial mediation model. The test result Δχ²(3) = 42.17, p < 0.001 indicates the full mediation model has significantly worse data fit. We reject the full mediation hypothesis and statistically confirm trust is a partial mediator.
   All test procedures, coefficient values and confidence intervals are supplemented in the results section, eliminating descriptive-only unsubstantiated claims.

**Editor Comment 4: The theoretical framework Figure 1 is mentioned but lacks systematic explanation of directional causal paths between all constructs**

**Response**

Two targeted revisions are implemented:

1. We created a publication-standard TIFF figure (Fig1.tif) following PLOS ONE image requirements. The figure clearly marks independent variables, mediator, moderator, dependent variable and labels each direct, indirect and moderating path corresponding to Hypotheses H1–H11.
2. A dedicated subsection “Theoretical Rationale for All Model Paths” is added at the beginning of Chapter 3 Research Hypotheses. We systematically interpret the causal logic of every single path supported by classic theories:

- Three service quality dimensions positively predict trust: Trust Theory (Gefen et al., 2003) distinguishes cognitive trust (streamer professionalism, after-sales support) and emotional trust (interactive experience);
- Trust positively predicts repurchase intention: Commitment-Trust Theory explains risk reduction and loyalty formation logic;
- Generational differences moderate service quality-trust links: Intergenerational Consumption Theory differentiates digital natives (Gen Z) and digital immigrants (older adults).
  Every directional hypothesis is grounded in seminal cited literature, fully resolving insufficient theoretical justification for the model structure.

**Part 2 Compliance with PLOS ONE Mandatory Submission Rules**

We strictly fulfill all journal administrative requirements listed in the decision letter:

1. Manuscript formatting: The full text is adjusted to match PLOS official templates (double-spaced, continuous line numbers, maximum three-level headings, sentence-case titles, Vancouver sequential reference style, tables/figures placed right after their first textual citation). Title page and main text follow the two official PDF format guides precisely.
2. Data Availability Statement: A standardized statement is placed in the manuscript and online submission form: All original questionnaire data, Mplus syntax files and output statistical results are deposited in the Dryad open repository with permanent DOI, fully accessible without access restrictions.
3. Ethics Statement: All ethics descriptions are removed from abstract, introduction and conclusion; we integrate and expand the full ethics paragraph solely within the “Research Design and Data Collection” subsection as PLOS mandates.
4. Reference standardization: All citations are cross-checked to exclude retracted articles. Every entry contains complete page numbers, standard journal abbreviations and consistent Vancouver sequential format. All literature recommended by reviewers is integrated and cited in relevant positions.
5. Figure specifications: All visual files are exported as 300 DPI TIFF images; captions are embedded in the main text rather than image files. Multi-panel layout rules are followed, and resolution limits are pre-checked.

**Part 3 Point-by-Point Responses to Individual Reviewers**

**Response to Reviewer #1**

**Comment 1: Severe content repetition between Theoretical Literature Review and Research Hypotheses sections**

**Response**

We restructured the two chapters to separate their functional positioning and delete redundant text:

1. Literature Review: Only sorts foundational theories, core construct definitions and universal empirical consensus, without repeated hypothesis-specific reasoning.
2. Research Hypotheses: Eliminates redundant literature summaries. Each hypothesis is logically deduced based on prior theoretical groundwork, with only concise targeted citations retained.

**Comment 2: Theoretical framework Figure 1 is missing in the original manuscript**

**Response**

A detailed figure (Fig1) caption explaining all paths and matching hypotheses is added in the manuscript, and the figure is cited immediately after the theoretical framework subsection.

**Comment 3: 88% valid response rate is abnormally high for online surveys; sample exclusion standards are vague and lack transparency**

**Response**

A new dedicated subsection “Questionnaire Exclusion Criteria” is added to the data collection chapter with quantifiable, pre-determined screening rules:

1. Minimum completion time threshold: Questionnaires finished within less than 120 seconds are eliminated (pre-test average valid filling time = 310s, short completion indicates careless random responses).
2. Straight-lining exclusion: Forms with identical 7-point Likert answers for over 12 consecutive items are discarded.
3. Logical inconsistency screening: Contradictory demographic-behavior combinations (e.g., self-reported Gen Z aged 18–24 but selecting elderly-oriented goods as main purchases) are excluded.
   We add breakdown statistics: 41 forms eliminated for short completion, 27 for straight-lining, 14 for logical conflict (82 invalid total). A supplementary robustness test result is also supplemented: we re-run all SEM models with borderline questionnaires retained, and core path coefficients show no substantive changes, proving sample stability after cleaning.

**Response to Reviewer #2**

**Comment 1: Original title is overly lengthy and requires simplification**

**Response**

Revised concise title:
Full title: Service Quality and Repurchase Intention in Chinese Live Commerce: The Mediating Role of Trust and the Moderating Effect of Generational Differences
Running head: Live commerce service quality and generational repurchase intention differences

**Comment 2: Introduction fails to clearly highlight the paper’s novel contributions against existing research**

**Response**

The final paragraph of the Introduction is fully rewritten to explicitly list three distinct theoretical and empirical innovations, with direct contrast against gaps identified in prior literature, making study novelty straightforward to identify for readers.

**Comment 3: Excessively long complex sentences; full-text linguistic polishing required**

**Response**

We completed full professional English revision: all lengthy multi-clause sentences are split into concise academic statements; overused passive voice is adjusted; ambiguous expressions and all typographical/grammatical errors are fixed. All linguistic edits are marked with track changes.

**Comment 4: Inconsistent sentence structure across Hypotheses H1–H11**

**Response**

All hypotheses are unified under identical parallel grammatical templates:

- Direct effects (H1–H7): [Independent variable] has a significant positive effect on [dependent variable].
- Mediation hypotheses (H8a–H8c): Trust partially mediates the relationship between each service quality dimension and repurchase intention.
- Moderation hypotheses (H9–H11): Generational differences significantly moderate the positive service quality-to-trust link.

**Comment 5: Table formatting needs unified optimization per journal standards**

**Response**

All tables are reconstructed as standard three-line tables following PLOS rules: simplified captions, unified two decimal places for all coefficients, redundant auxiliary notes removed, consistent text alignment. Table citations in text are reordered sequentially.

**Comment 6: Terminology inconsistency: unify “elderly / older adults”**

**Response**

The neutral standardized term *older adults* is adopted uniformly throughout the manuscript; all scattered “elderly” expressions are fully replaced without mixed wording.

**Comment 7: Full manuscript proofreading required**

**Response**

We double-proofread the entire text, including paragraphs, tables, figure captions and reference lists, to fix typos, inconsistent statistical symbols and citation mismatches.

**Response to Reviewer #3**

**Comment 1: Theoretical architecture lacks sufficient grounding; strengthen core theoretical basis**

**Response**

We adopt the IMPACT framework (Hollebeek et al., 2025) recommended by the reviewer to rationalize theoretical selection. Trust Theory serves as the core overarching framework, while revised SERVQUAL and Intergenerational Consumption Theory act as auxiliary supporting theories. A full IMPACT criterion analysis is integrated into the theoretical foundation subsection as suggested.

**Comment 2: Deepen multi-dimensional operationalization of live commerce service quality**

**Response**

We expand the discussion of the three service quality dimensions and link each to interactivity, informativeness and reliability sub-facets specific to live streaming e-commerce. Supplementary Table S1 is added to list all sub-items under streamer professionalism, interactive experience and after-sales support to clarify multi-dimensional measurement design.

**Comment 3: Optimize stratified sampling description and sample sufficiency for multi-group SEM**

**Response**

The sampling section is expanded to detail stratified quota sampling across eastern, southern, northern and western China, plus Douyin/Taobao/Kuaishou platform stratification, strictly balancing 300 Gen Z and 300 respondents in the older adult group. We add power analysis results: per Kline (2023), n=30 per subgroup far exceeds the minimum sample threshold for stable multi-group moderated mediation path estimation.

**Response to Reviewer #5**

**Comment 1: Adopt the IMPACT framework to rationalize theory selection and cite Hollebeek et al. (2025)**

**Response**

We fully integrate the IMPACT standard analysis into the theoretical foundation chapter as suggested. We systematically evaluate the suitability of Trust Theory as the core framework and the auxiliary value of revised SERVQUAL and intergenerational consumption theories from six dimensions (Interestingness, Matching, Parsimony, Applicability, Conceptual Rigor, Testability). Hollebeek et al. (2025) is formally cited in-text and added to the reference list with complete DOI.

**Comment 2: Clarify theoretical novelty using Lim (2026b) theory development typology**

**Response**

The “Theoretical Contributions” subsection in Chapter 6 is rewritten following Lim (2026b). We classify innovations into two categories: (1) theoretical adaptation: reconstruct SERVQUAL into live-commerce-specific three dimensions; (2) theoretical extension: identify generational cohort as a boundary variable altering trust mediation strength. We elaborate the biases caused by ignoring these gaps in prior research and explain how this paper resolves ambiguous trust transmission mechanisms across age groups. Lim (2026b) is fully referenced.

**Comment 3: Restrict intention-based conclusions with the 4Cs framework (Lim, 2026a)**

**Response**

A standalone limitation paragraph based on the 4Cs (Claim, Correspondence, Constraint, Corroboration) framework is added to Section 6.4. We clearly distinguish subjective repurchase intention from objective real transaction behavior, list interfering variables (price fluctuation, product stock, platform switching cost, household joint purchase) that block intention-behavior conversion, and propose longitudinal transaction data collection for follow-up research. Lim (2026a) is added to references.

**Comment 4: Clarify reflective second-order operationalization of the trust construct**

**Response**

We resolve the reviewer’s confusion about single vs dual trust dimensions in the measurement section and CFA results:

1. Theoretically, this paper differentiates cognitive and emotional trust; empirically, all scale items converge into a unified second-order reflective trust latent variable.
2. Supplementary Table S2 is added to compare single-factor vs two-factor trust model fit indices, proving the unified trust construct has better matching performance for this sample.
3. We explicitly explain in the text that cognitive/emotional division is used for theoretical reasoning, while the unified second-order trust variable is adopted for SEM path testing.

**Comment 5: Add measurement invariance testing before multi-group comparison; fix Table 11 duplicate H8c typo**

**Response**

1. A complete three-stage measurement invariance test (configural, metric, scalar) is supplemented before Section 5.8 Moderating Effect Testing, with dedicated Table 12 attached. All ΔCFI and ΔRMSEA values meet standard cutoffs, proving cross-group measurement equivalence and validating subsequent structural path comparison.
2. The duplicate H8c label error in Table 11 is corrected; all mediation hypothesis numbering is standardized without repetition.

**Comment 6: Unreliable market sources, inconsistent data & ethics descriptions**

**Response**

All related revisions are fully implemented as addressed in Editor Comment 1 and Comment 2. Market statistics are updated to official government reports; data availability and ethics paragraphs are harmonized to eliminate contradictory statements across the manuscript and submission system.

**Comment 7: Practical implications are overly generalized; link recommendations to path coefficients**

**Response**

Chapter 6.3 Practical Implications is fully restructured with quantitative coefficient support:

- Gen Z priority: Interactive experience (β=0.43) – develop AR fitting, live gamification and real-time comment interaction tools as core investment;
- Older adults priorities: Streamer professionalism (β=0.37) + after-sales support (β=0.32) – launch streamer qualification certification, dedicated manual customer service and simplified broadcast after-sales pop-ups;
  All operational strategies are directly paired with corresponding multi-group standardized path results to provide quantifiable, actionable guidance for live commerce operators.

**Response to Reviewer #4**

The reviewer acknowledged the manuscript’s sound structure, rigorous methodology and reasonable sample exclusion rules, and raised no additional revision requests. We sincerely appreciate the positive evaluation of our research.

We sincerely thank the editorial team and all reviewers for their careful guidance and valuable suggestions. We will strictly follow all journal production requirements if this manuscript is accepted.
