# Supplementary material for: Service quality and repurchase intention in Chinese live commerce: The mediating role of trust and the moderating effect of generational differences
Source: PLoS One. 2026 Aug 26;21(8):e0356797. doi: 10.1371/journal.pone.0356797 (PMC13514050; doi:10.1371/journal.pone.0356797)
Supplement: Peer review history

# Peer review: Decision Letter 0

**DOI:** 10.1371/journal.pone.0356797.r001

**Authors:** Sudarsan Jayasingh

5 Jun 2026

Dear Dr. He,

Thank you for submitting your manuscript to PLOS ONE. After careful consideration, we feel that it has merit but does not fully meet PLOS ONE’s publication criteria as it currently stands. Therefore, we invite you to submit a revised version of the manuscript that addresses the points raised during the review process.

Please submit your revised manuscript by Jul 20 2026 11:59PM. If you will need more time than this to complete your revisions, please reply to this message or contact the journal office at plosone@plos.org. When you're ready to submit your revision, log on to https://www.editorialmanager.com/pone/ and select the 'Submissions Needing Revision' folder to locate your manuscript file.

- A letter that responds to each point raised by the academic editor and reviewer(s). You should upload this letter as a separate file labeled 'Response to Reviewers'.
- A marked-up copy of your manuscript that highlights changes made to the original version. You should upload this as a separate file labeled 'Revised Manuscript with Track Changes'.
- An unmarked version of your revised paper without tracked changes. You should upload this as a separate file labeled 'Manuscript'.

If you would like to make changes to your financial disclosure, please include your updated statement in your cover letter. Guidelines for resubmitting your figure files are available below the reviewer comments at the end of this letter.

If applicable, we recommend that you deposit your laboratory protocols in protocols.io to enhance the reproducibility of your results. Protocols.io assigns your protocol its own identifier (DOI) so that it can be cited independently in the future. For instructions see: https://journals.plos.org/plosone/s/submission-guidelines#loc-laboratory-protocols. Additionally, PLOS ONE offers an option for publishing peer-reviewed Lab Protocol articles, which describe protocols hosted on protocols.io. Read more information on sharing protocols at https://plos.org/protocols?utm_medium=editorial-email&utm_source=authorletters&utm_campaign=protocols.

As the corresponding author, your ORCID iD is verified in the submission system and will appear in the published article. PLOS supports the use of ORCID, and we encourage all coauthors to register for an ORCID iD and use it as well. Please encourage your coauthors to verify their ORCID iD within the submission system before final acceptance, as unverified ORCID iDs will not appear in the published article. *Only*  the individual author can complete the verification step; PLOS staff *cannot*  verify ORCID iDs on behalf of authors.

We look forward to receiving your revised manuscript.

Kind regards,

Sudarsan Jayasingh, Ph.D

Academic Editor

PLOS One

**Journal Requirements:**

When submitting your revision, we need you to address these additional requirements.

1. Please ensure that your manuscript meets PLOS ONE's style requirements, including those for file naming. The PLOS ONE style templates can be found at

https://journals.plos.org/plosone/s/file?id=wjVg/PLOSOne_formatting_sample_main_body.pdf and

https://journals.plos.org/plosone/s/file?id=ba62/PLOSOne_formatting_sample_title_authors_affiliations.pdf

2. Please provide a complete Data Availability Statement in the submission form, ensuring you include all necessary access information or a reason for why you are unable to make your data freely accessible. If your research concerns only data provided within your submission, please write "All data are in the manuscript and/or supporting information files" as your Data Availability Statement.

3. Your ethics statement should only appear in the Methods section of your manuscript. If your ethics statement is written in any section besides the Methods, please move it to the Methods section and delete it from any other section. Please ensure that your ethics statement is included in your manuscript, as the ethics statement entered into the online submission form will not be published alongside your manuscript.

4. If the reviewer comments include a recommendation to cite specific previously published works, please review and evaluate these publications to determine whether they are relevant and should be cited. There is no requirement to cite these works unless the editor has indicated otherwise.

5. Please review your reference list to ensure that it is complete and correct. If you have cited papers that have been retracted, please include the rationale for doing so in the manuscript text, or remove these references and replace them with relevant current references. Any changes to the reference list should be mentioned in the rebuttal letter that accompanies your revised manuscript. If you need to cite a retracted article, indicate the article’s retracted status in the References list and also include a citation and full reference for the retraction notice.

**Additional Editor Comments:**

1. "Renrendoc" are cited as sources for market statistics - they are not primary data sources and should not be treated as citable research. Replacing non-academic sources (Renrendoc, Originality Documents) with credible references.

2. Line 70 states that China's live commerce user base "had exceeded 8.2 billion" by 2024. This figure is impossible — the entire global population is approximately 8 billion.

3. The authors claim trust "partially mediates" the service quality–repurchase intention relationship, but they do not formally test partial vs. full mediation.

4. The theoretical framework figure is referenced; however, the rationale for the directional relationships among the constructs is not sufficiently explained.

[Note: HTML markup is below. Please do not edit.]

Reviewers' comments:

Reviewer's Responses to Questions

**Comments to the Author**

1. Is the manuscript technically sound, and do the data support the conclusions?

Reviewer #1: Yes

Reviewer #2: Yes

Reviewer #3: Yes

Reviewer #4: Yes

Reviewer #5: Yes

**********

2. Has the statistical analysis been performed appropriately and rigorously?

Reviewer #1: Yes

Reviewer #2: Yes

Reviewer #3: Yes

Reviewer #4: Yes

Reviewer #5: Yes

**********

3. Have the authors made all data underlying the findings in their manuscript fully available?

The PLOS Data policy

Reviewer #1: Yes

Reviewer #2: No

Reviewer #3: Yes

Reviewer #4: Yes

Reviewer #5: Yes

**********

4. Is the manuscript presented in an intelligible fashion and written in standard English?

Reviewer #1: Yes

Reviewer #2: Yes

Reviewer #3: Yes

Reviewer #4: Yes

Reviewer #5: Yes

**********

Reviewer #1: This study addresses an important and current topic with a sound methodological approach. The core findings are interesting and have practical value.

1. Repetitive Literature Review: The "Theoretical Foundation and Literature Review" and "Research Hypotheses" sections are highly repetitive. The literature review introduces the core variables and their relationships, only for the hypotheses section to re-introduce the same literature and arguments in nearly identical language.

2. Figure 1 (Theoretical Framework) is Missing. A visual representation of the model is essential for a paper of this complexity. Its absence makes it difficult for the reader to follow the proposed relationships (H1-H11). This must be added.

3. The manuscript reports an "88% effective response rate" after excluding 82 of 682 questionnaires for "short completion time, homogeneous answers, logical inconsistencies." An 88% validity rate is exceptionally high for an online survey, and the exclusion criteria are somewhat subjective. Was there a specific time cutoff used? What constituted a "logical inconsistency"? Providing more detail on these exclusion rules would strengthen the transparency and replicability of the study.

Reviewer #2: Availability of data, the title is long and needs reformulation, reformulation of the introduction to be clearer about what the new addition is, revision of long sentences we recommend a simple linguistic revision, formulation of hypotheses in a more consistent way, improved presentation of tables, we recommend standardization of terminology (elderly, for example) and proofreading.

Reviewer #3: The study can be strengthened by refining both its theoretical grounding and methodological rigor. First, the researcher should anchor the model in a clear theoretical framework to better justify the relationships between service quality, trust, and repurchase intention. It is also recommended to operationalize live commerce service quality as a multidimensional construct (e.g., interactivity, responsiveness, informativeness, and entertainment) to enhance analytical depth. From a methodological perspective, the researcher could improve robustness by adopting a stratified sampling approach to ensure balanced representation between Gen Z and older consumers, and by increasing sample size to support multi-group analysis.

Reviewer #4: The paper is well written and well thought out and provides insight into an interesting area. All areas that have a potential issues have been adequately addressed including the exclusion of the other groups in the study. Based on this my verdict is to accept the paper.

Reviewer #5: This paper explores how live commerce service quality shapes repurchase intention in China through trust, with generational differences between Generation Z and older adults. The idea is interesting and relevant, particularly as live commerce platforms need more precise strategies for users who form trust through different service cues. I do, however, have several observations and suggestions for improvement.

First, the study already refers to trust theory, a modified service quality model, and generational consumption logic, but the theoretical foundation still reads more like a collection of related frameworks than a clearly justified theoretical architecture. This is an issue as the paper’s explanatory value depends on showing why the selected theory is the best fit for the focal mechanism, rather than only showing that prior studies have used related constructs. To address this issue, please ground the paper more explicitly in a primary theory, with other frameworks positioned as supporting measurement or boundary-condition lenses. A good way to do this is to use and report the coherence of the selected theory with the IMPACT criteria for theory selection (see and leverage the article “Guidelines for theory selection: The IMPACT framework”). You may consider saying something along the following lines after the paragraph where the paper introduces its three frameworks:

“Guided by the IMPACT framework for theory selection (interestingness, matching, parsimony, applicability, conceptual rigor, and testability; Hollebeek et al., 2025), this study positions trust theory as the primary explanatory lens and the adapted service quality model as the measurement architecture, whereby interestingness lies in explaining why the same service cues do not generate uniform repurchase intention across generational groups, whereas matching is strong as the focal mechanism concerns trust formation under uncertainty in live commerce exchanges while parsimony is reflected in a single mediating mechanism, trust, linking service cues to repurchase intention, applicability lies in translating trust mechanisms into platform design choices for Generation Z and older adults, conceptual rigor is supported through the explicit linkage among service cues, trust and intention, and testability is reflected in the moderated mediation model that compares the strength of service quality to trust relationships across generational groups.”

Supporting reference:

Guidelines for theory selection: The IMPACT framework. Psychology & Marketing.

Second, the theoretical gaps and contributions need to be problematized more sharply. The current version states that prior studies have overlooked trust as a mediator and generational differences as a boundary condition, but the paper should explain why these omissions limit current understanding of live commerce and what theoretical misunderstanding may arise without addressing them. This is an issue as novelty cannot rest on the argument of limited research alone. To address this issue, please specify the exact extent of theoretical novelty and the type of theoretical interestingness that the study offers with support from extant guidance on theory development (see and leverage the article “Theory and theory development: Guidelines for establishing theoretical gaps, foundations, contributions, and implications”). You may consider saying something along the following lines at the end of the introduction or at the start of the theoretical contributions section:

“In line with extant typologies of theory development (Lim, 2026b), the theoretical contribution of this study can be positioned as an incremental but useful theoretical adaptation and extension. The theoretical adaptation lies in tailoring service quality to live commerce through streamer professionalism, interactive experience, and after-sales support, whereas the theoretical extension lies in showing that trust does not operate as a uniform mediator across generations. Notably, Generation Z appears to form trust more strongly through interactive experience, whereas older adults appear to form trust more strongly through professionalism and after-sales support. Such an insight is theoretically noteworthy as the same service quality architecture produces different trust pathways across age-based digital experience profiles, which clarifies the boundary conditions under which service quality converts into repurchase intention.”

Supporting reference:

Theory and theory development: Guidelines for establishing theoretical gaps, foundations, contributions, and implications. Journal of Business Research.

Third, the paper uses repurchase intention as the outcome, which is acceptable, but the claims need to be bounded more carefully. This is an issue as intention evidence can support psychological readiness or willingness, but it cannot directly support claims about actual repurchase behavior, customer retention, or sales performance unless behavioral evidence is collected. To address this issue, please add a limitation that uses the 4Cs of intention research, namely claim, correspondence, constraint and corroboration (see and leverage the article “Guidelines for intention research”). You may consider adding the following to the limitations section:

“As this study examines repurchase intention rather than observed repurchase behavior, claims should remain psychological rather than behavioral. The present evidence can support claims about willingness to repurchase and the perceived conditions under which trust strengthens such willingness. The evidence cannot yet support claims that consumers will actually repurchase or that platform interventions will increase repeat sales without behavioral corroboration. Following the 4Cs framework (claim, correspondence, constraint, and corroborate; Lim, 2026a), future research should strengthen claim alignment, improve correspondence between target, action, context and time window, assess constraints that may block enactment, such as price, stock availability, platform switching and family influence, and corroborate intention findings with transaction records or longitudinal follow-up data.”

Supporting reference:

Guidelines for intention research. Annals of Tourism Research.

Fourth, the measurement of trust needs clearer conceptual handling. The paper labels trust as cognitive and emotional trust, but the analysis appears to treat trust as a single construct. This creates ambiguity as cognitive trust, emotional trust, streamer trust and platform trust may not be interchangeable. To address this issue, please clarify whether trust is modeled as a single reflective construct, a second-order construct, or two separate dimensions. The paper should also explain why streamer trust and platform trust can be combined, or report additional tests showing that they are empirically and theoretically coherent as one construct.

Fifth, the generational comparison needs stronger methodological support. The paper compares Generation Z and older adults, but such comparisons require evidence that both groups interpret the constructs in sufficiently similar ways before differences in relationships are interpreted. To address this issue, please report measurement invariance tests before the multi-group comparison. At minimum, the paper should explain whether the measurement model is comparable across groups, including whether factor loadings are stable enough to justify comparing structural paths. The paper should also correct reporting inconsistencies, such as the repeated label for H8c in the mediation table, since such small errors can reduce confidence in an otherwise useful analysis.

Sixth, the paper should strengthen source credibility and reporting precision. Some market statistics rely heavily on industry sources such as Originality Documents, Renrendoc, Docin and GetShogun, and one claim that China’s live commerce user base exceeded 8.2 billion (that’s more than the world population!) appears implausible and likely reflects a unit or translation error. To address this issue, please verify all market figures against authoritative sources, correct units where needed, and replace weak or nontransparent sources with traceable government, platform, industry or peer-reviewed evidence. The data availability and ethics statements should also be harmonized, especially where the paper states that data were uploaded to the submission system and elsewhere states that raw data will be made available.

Seventh, the implications should be more tightly tied to the empirical findings. The current recommendations, such as augmented reality try-ons for Generation Z and human-centered after-sales support for older adults, are sensible but still broad. This is an issue as the paper’s practical value lies in helping platforms decide which service investments should be prioritized for which user segment. To address this issue, please translate the coefficients into clearer managerial priorities. For example, platforms targeting Generation Z can prioritize low-friction interaction design, real-time participation and engaging product demonstrations, whereas platforms targeting older adults can prioritize streamer certification, transparent return policies and human after-sales support. This would make the implications more actionable and better aligned with the moderated mediation findings.

I hope these comments will be useful to improve the quality of this work.

I look forward to reading a well-revised version.

Good luck and all the very best!

**********

what does this mean?). If published, this will include your full peer review and any attached files.

If you choose “no”, your identity will remain anonymous but your review may still be made public.

**Do you want your identity to be public for this peer review?**  For information about this choice, including consent withdrawal, please see our Privacy Policy

Reviewer #1: No

Reviewer #2: No

Reviewer #3: No

Reviewer #4: No

Reviewer #5: No

**********

[NOTE: If reviewer comments were submitted as an attachment file, they will be attached to this email and accessible via the submission site. Please log into your account, locate the manuscript record, and check for the action link "View Attachments". If this link does not appear, there are no attachment files.]

To ensure your figures meet our technical requirements, please review our figure guidelines: https://journals.plos.org/plosone/s/figures

You may also use PLOS’s free figure tool, NAAS, to help you prepare publication quality figures: https://journals.plos.org/plosone/s/figures#loc-tools-for-figure-preparation.

NAAS will assess whether your figures meet our technical requirements by comparing each figure against our figure specifications.

# Author comment: Author response to Decision Letter 1

**DOI:** 10.1371/journal.pone.0356797.r002

20 Jul 2026

We sincerely thank the Academic Editor and all independent reviewers for their rigorous, detailed, and constructive feedback on our manuscript. Every editorial and reviewer comment has been fully addressed with targeted revisions. All textual, structural, statistical, formatting and ethical adjustments are tracked with revision marks in the uploaded revised manuscript. All changes strictly adhere to PLOS ONE official formatting, data transparency, ethics and citation standards.

Attachment Submitted filename: Response to Reviewers.docx

# Editor report: Decision Letter 1

**DOI:** 10.1371/journal.pone.0356797.r003

**Authors:** Sudarsan Jayasingh

10 Aug 2026

Service Quality and Repurchase Intention in Chinese Live Commerce: The Mediating Role of Trust and the Moderating Effect of Generational Differences

PONE-D-26-06405R1

Dear Dr. He,

We’re pleased to inform you that your manuscript has been judged scientifically suitable for publication and will be formally accepted for publication once it meets all outstanding technical requirements.

Within one week, you’ll receive an e-mail detailing the required amendments. When these have been addressed, you’ll receive a formal acceptance letter and your manuscript will be scheduled for publication.

An invoice will be generated when your article is formally accepted. Please note, if your institution has a publishing partnership with PLOS and your article meets the relevant criteria, all or part of your publication costs will be covered. Please make sure your user information is up-to-date by logging into Editorial Manager at Editorial Manager® and clicking the ‘Update My Information' link at the top of the page. For questions related to billing, please contact billing support.

If your institution or institutions have a press office, please notify them about your upcoming paper to help maximize its impact. If they’ll be preparing press materials, please inform our press team as soon as possible -- no later than 48 hours after receiving the formal acceptance. Your manuscript will remain under strict press embargo until 2 pm Eastern Time on the date of publication. For more information, please contact onepress@plos.org.

Kind regards,

Sudarsan Jayasingh, Ph.D

Academic Editor

PLOS One

Additional Editor Comments (optional):

Reviewers' comments:

# Editor report: Acceptance letter

**DOI:** 10.1371/journal.pone.0356797.r004

**Authors:** Sudarsan Jayasingh

PONE-D-26-06405R1

PLOS One

Dear Dr. He,

I'm pleased to inform you that your manuscript has been deemed suitable for publication in PLOS One. Congratulations! Your manuscript is now being handed over to our production team.

At this stage, our production department will prepare your paper for publication. This includes ensuring the following:

* All references, tables, and figures are properly cited

* All relevant supporting information is included in the manuscript submission,

* There are no issues that prevent the paper from being properly typeset

You will receive further instructions from the production team, including instructions on how to review your proof when it is ready. Please keep in mind that we are working through a large volume of accepted articles, so please give us a few days to review your paper and let you know the next and final steps.

Lastly, if your institution or institutions have a press office, please let them know about your upcoming paper now to help maximize its impact. If they'll be preparing press materials, please inform our press team within the next 48 hours. Your manuscript will remain under strict press embargo until 2 pm Eastern Time on the date of publication. For more information, please contact onepress@plos.org.

You will receive an invoice from PLOS for your publication fee after your manuscript has reached the completed accept phase. If you receive an email requesting payment before acceptance or for any other service, this may be a phishing scheme. Learn how to identify phishing emails and protect your accounts at https://explore.plos.org/phishing.

If we can help with anything else, please email us at customercare@plos.org.

Thank you for submitting your work to PLOS One and supporting open access.

Kind regards,

PLOS ONE Editorial Office Staff

on behalf of

Dr. Sudarsan Jayasingh

Academic Editor

PLOS One
